# Wildfire disruptions and cancer care: A multi-institutional study of radiotherapy treatment adherence in Northern California

**DOI:** 10.1016/j.joclim.2025.100530

**Published:** 2025-10-03

**Authors:** R.A. Sabol, C.J. Walsh, S. Densley, Y. Medhat, C.C. Baniel, A. Krishna, C.T. Baiyee, D. Meltzer, J. Boscardin, A. Witztum, N. Pitts, A.K. Paulsson, J.Y. Luh, L. Zalavari, S.S. Yom, K. Lichter

**Affiliations:** a Department of Radiation Oncology, University of California San Francisco, San Francisco, CA, USA; b University of Colorado School of Medicine, Aurora, CO, USA; c Florida Atlantic University College of Medicine, Boca Raton, FL, USA; d University of California, San Francisco (UCSF), San Francisco, CA, USA; e Stanford HealthCare, Palo Alto, CA, USA; f Tufts Medical Center, Boston, MA, USA; g Ohio State College of Medicine, Columbus, OH, USA; h Providence Medical Group Northern California, Petaluma, CA, USA; i Providence St. Joseph Health, Eureka, CA, USA; j Department of Radiation Oncology and Applied Sciences, Dartmouth Cancer Center, NH, Lebanon; k The Dartmouth Institute, Geisel School of Medicine, NH, Lebanon

**Keywords:** Wildfire, Climate change, Radiotherapy, Cancer treatment, Climate disruption, Climate health

## Abstract

**Introduction:**

Climate-driven disasters such as wildfires are increasing in both severity and frequency, posing serious threats to healthcare infrastructure, continuity of care, and patient well-being. Individuals undergoing cancer treatment are particularly vulnerable, as care often requires frequent and time-sensitive visits over several weeks to specific treatment centers—especially in the case of radiotherapy. However, the impact of climate-related wildfires on radiotherapy treatment adherence remains largely unexplored.

**Methods:**

This multi-institutional retrospective cohort analyzed 539,292 radiotherapy treatment appointments from eight clinics in Northern California between 2017–2021 and compared rates of missed visits during active wildfires. Wildfire data from the California Department of Forestry and Fire Protection (CAL FIRE) were used to correlate the proximity of wildfires (within a 50-kilometer [km] radius) to clinics. Missed visit rates were compared between treatment visits that coincided with active wildfires within 50-km and those that did not.

**Results:**

Overall, 8.8 % of appointments coincided with wildfires within a 50-km radius, and 4.9 % of these were missed. Wildfire exposure was associated with increased missed appointments (OR = 1.07, 95 % CI [1.02, 1.13], *p* = 0.007) after adjusting for seasonality, temporal trends, and clinic factors. Demographic analyses revealed no significant patient-specific disparities in missed treatments. The estimated financial impact of missed treatments was $2.14 million, highlighting economic vulnerabilities.

**Conclusion:**

This initial investigation demonstrates a statistically significant trend towards higher non-adherence to radiotherapy visits during wildfires. This is the first multi-institutional study to investigate the impact of wildfires exposure on radiotherapy adherence and to quantify the associated financial impact. Our findings highlight the emerging intersection of climate change and cancer care delivery, emphasizing the need for health system resilience in the face of environmental threats.

## 1 Introduction

The increasing frequency and severity of climate-driven disasters—such as wildfires, hurricanes, flooding, and heatwaves—pose significant threats to healthcare infrastructure, patient safety, and continuity of care [1,2]. Oncology patients, particularly those receiving time-sensitive treatments like radiotherapy, are uniquely vulnerable to these disruptions. Unfortunately, adherence to care protocols during disasters has not been systematically quantified, leaving a critical gap in health system preparedness and policy response [3].

Addressing this gap is essential for developing data-driven policies that protect oncology patients, healthcare operations, and financial stability. Radiotherapy requires consistent, daily treatments, and interruptions have been linked to tumor progression and reduced survival [4,5]. Yet, there are no established national strategies to ensure radiotherapy and cancer care continuity during climate-related crises [3]. This multi-institutional study quantifies wildfire-related radiotherapy disruptions to inform policy solutions that enhance healthcare resilience and equity during climate disasters.

## 2 Methods

**Fig. 1:**
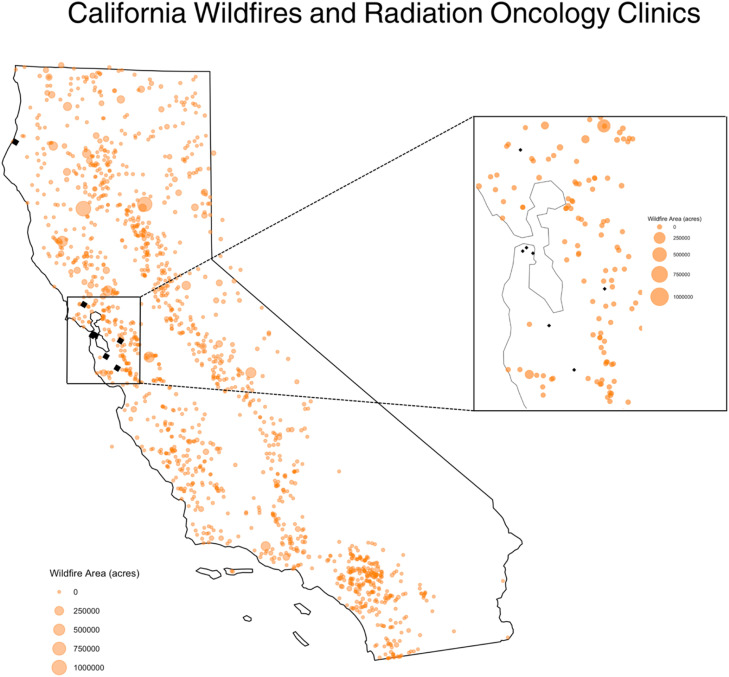
Geography of California wildfires and radiation oncology clinics. Approximate locations of eight Northern California radiation oncology clinics (represented as black diamonds) in relation to the 2017 to 2021 wildfires per CAL FIRE wildfire data with wildfire area burned (acres) represented per graphical figure legend.

This retrospective cohort study analyzed 539,292 radiotherapy treatment appointments across eight clinics in California from 2017 to 2021, encompassing varied clinical settings including academic and community-based centers in urban (7 sites) and rural (1 site) locations based on the Federal Office of Rural Health Policy defined rural areas. Wildfire data, including location, start date, and date of containment of wildfires, was acquired from the California Department of Forestry and Fire Protection (CAL FIRE) and were used to correlate appointments that took place during wildfires and assess the proximity of wildfires (within a 50-km [km] radius) to clinics (Fig. 1) [6]. Univariable and multivariable logistic regressions were performed to account for baseline variation in missed visits based on time of year, changes over time across the study period of 2017–2021, and location. Analysis of associations between missed treatments and patient characteristics including age, sex, diagnosis, and race were performed. Analyses were conducted using Stata 18.0 and R 4.2.3. In order to estimate the financial impact of missed radiotherapy treatments, the average cost per radiotherapy treatment appointment was acquired from a single institution. The average cost was generated from by combining average labor and non-labor costs per treatment visit. This was then multiplied by the number of missed visits during times when a wildfire was active within 50-km of clinic to generate an estimate of the financial impact of missed appointments during times of active wildfires.

## 3 Results

This study included 539,292 radiotherapy appointments, of which 8.8 % coincided with wildfires occurring within a 50-km radius of clinics and 4.9 % of these appointments were missed (Table 1). Univariable analysis revealed a modest but statistically significant increase in missed appointments during wildfires (OR = 1.06, 95 % CI [1.00, 1.11], *p* = 0.039), with a more pronounced effect in the multivariable analysis adjusting for seasonality, temporal trends, and clinic location (OR = 1.07 [1.02, 1.13], *p* = 0.007). The adjusted missed appointment rate during wildfires was 5.2%, compared to 4.8 % in the absence of wildfires. Of note, in comparing the missed visit rate at individual clinical sites, three sites had, by percentage, lower rates of missed visits during times of active wildfires. However, chi-square analysis demonstrated that this was not a statistically significant difference in missed visits. In an analysis utilizing demographic patient data from a single institution, there was no significant between missed visit rate and patient characteristics (including age, sex, diagnosis, or race). Based on the cost analysis derived from the data of one institution, the estimated financial impact of missed treatments due to wildfire disruptions at all of the facilities was estimated as $2.14 million.

**Table 1:** Missed radiotherapy treatment appointments. This table summarizes the number of missed radiotherapy treatment appointments at each de-identified clinical site. Missed visit number and rate for when no wildfires were active within 50-kilometers (km), when wildfires were active within a 50-km radius, and total number of missed treatment appointments for each clinical site and total during the study period.

Clinical Site	Number and rate (%) of missed visits when no wildfires are active within 50-km (*n* = 491,989)	Number and rate (%) of missed visits when there are active wildfires within 50-km (*n* = 47,303)	Total number and rate (%) of missed visits (*n* = 539,292)
1	2937 (3.94 %)	429 (4.23 %)	3366 (3.98 %)
2	1952 (3.51 %)	119 (3.05 %)	2071 (3.48 %)
3	1627 (4.74 %)	110 (4.64 %)	1737 (4.73 %)
4	3591 (4.88 %)	252 (5.74 %)	3843 (4.93 %)
5	3557 (5.04 %)	358 (4.93 %)	3915 (5.03 %)
6	6753 (5.75 %)	721 (6.06 %)	7474 (5.80 %)
7	849 (4.96 %)	118 (5.60 %)	967 (5.03 %)
8	2610 (5.34 %)	308 (5.37 %)	2918 (5.35 %)
Overall	23,876 (4.85 %)	2415 (5.11 %)	26,291 (4.88 %)

## 4 Discussion

This study provides the first multi-institutional evidence that climate-fueled disasters disrupt cancer care by reducing radiotherapy adherence, reinforcing the urgent need for health policy interventions that strengthen system resilience. The observed increase in missed treatments during wildfires, though modest, represents a systemic vulnerability that could escalate as climate disasters intensify.

While not statistically significant, there were three clinics with decreased rates of missed visits during wildfires. This may represent a difference in vulnerabilities based on patient population, clinic location, or other factors. Multivariable analysis of seasonality and location as well as available demographic data from a single institution was performed and did not reveal significant associations between missed visits and patient age, sex, diagnosis or race; however, this study may have been underpowered to detect such vulnerabilities given the overall low rates of missed visits.

### 4.1 Policy and health system implications

• **Healthcare Infrastructure & Disaster Preparedness** – Federal and state agencies must integrate climate resilience planning into healthcare regulations, including updated accreditation requirements, contingency plans, and emergency transportation services.
• **Economic Impact & Financial Protections** – Missed treatments due to wildfires resulted in an estimated $2.14 million loss, reflecting broader economic risks to health systems and insurers. Policy solutions should include reimbursement safeguards for climate-related disruptions to protect oncology centers from revenue loss.
• **Equity & Patient Access** – These data demonstrate some variability in missed visits across clinic sites suggesting variability in vulnerabilities to wildfires exist, though multivariable analysis did not reveal a specific patient or clinic factor as significant. Nevertheless, development of infrastructure to mitigate climate-related treatment disruptions in clinics in regions susceptible to wildfires is encouraged to ensure equity and patient access. Examples of policies that could help reduce disparities in access during climate events could include expanding telemedicine services, mobile treatment units, and transportation assistance.

### 4.2 Conclusion

As climate change accelerates, future research should identify high-risk populations for treatment disruption, assess long-term clinical and economic consequences of climate-driven treatment delays, and inform data-driven policy frameworks to safeguard oncology care. In an era of increasing environmental instability, policymakers, healthcare systems, and researchers must collaborate to develop adaptive strategies that ensure uninterrupted access to critical cancer treatments.

## CRediT authorship contribution statement

**R.A. Sabol:** Writing – review & editing, Writing – original draft, Validation, Supervision, Project administration, Methodology, Investigation, Formal analysis, Conceptualization. **C.J. Walsh:** Writing – review & editing, Writing – original draft, Methodology, Data curation. **S. Densley:** Writing – review & editing, Writing – original draft, Investigation, Data curation. **Y. Medhat:** Writing – review & editing, Writing – original draft, Investigation, Data curation. **C.C. Baniel:** Writing – review & editing, Supervision, Investigation, Conceptualization. **A. Krishna:** Writing – review & editing, Methodology, Funding acquisition, Conceptualization. **C.T. Baiyee:** Writing – review & editing, Investigation, Conceptualization. **D. Meltzer:** Writing – review & editing, Validation, Software, Formal analysis. **J. Boscardin:** Writing – review & editing, Validation, Software, Methodology, Investigation, Formal analysis, Data curation. **A. Witztum:** Writing – review & editing, Validation, Software, Resources, Methodology, Data curation. **N. Pitts:** Writing – review & editing, Investigation, Data curation, Conceptualization. **A.K. Paulsson:** Writing – review & editing, Supervision, Methodology, Funding acquisition, Data curation, Conceptualization. **J.Y. Luh:** Writing – review & editing, Supervision, Methodology, Investigation, Data curation, Conceptualization. **L. Zalavari:** Writing – review & editing, Resources, Methodology, Investigation, Data curation, Conceptualization. **S.S. Yom:** Writing – review & editing, Supervision, Resources, Methodology, Investigation, Conceptualization. **K. Lichter:** Writing – review & editing, Methodology, Funding acquisition, Formal analysis, Data curation, Conceptualization.

## Declaration of competing interest

The authors declare the following financial interests/personal relationships which may be considered as potential competing interests: SSY – grants/contracts in the last 36 months from Merck, EDM Serono, Bristol-Myers Squibb and Nanobiotix paid to the authors institution for research support. Royalties/licenses in the last 36 months – UptoDate – Royalty paid to the author, Springer – Royalty paid to the author. Payment or honoraria for lecture, presentations, speakers, bureaus, manuscript writing or educational events – American Society of Radiation Oncology – honoraria paid to the author, Elsevier – honoraria paid to the author.
